# Management of Primary Dentition

**Published:** 1891-04

**Authors:** 


### Management of Primary Dentition.

According to Dr. Monti, *Journal de Sciences Medicale de Lille*, all interference with primary dentition should, as a general rule, be proscribed. Hence he considers as injurious the ordinary practices, as, for example, biting on hard objects, bathing the gums with so-called emollient substances, etc.

The only necessary measure of precaution is to keep the buccal mucous membrane in a state of perfect cleanliness. It is best, during dentition, to wash the mouth several times daily, either with pure water or antiseptic solutions. Among the latter, the most efficacious are the following:

R. Acidi boracici.........................gr. xiv.

Aquæ destillatæ......................ounces viii.

Tinct. myrrhæ.......................*m* xiv.—M.

Or,

R. Sodii salicylatis .......................gr. xiv.

Aquæ destillatæ......................ounces viii.

Tinct. myrrhæ......... ..............*m* xiv.—M.

When the milk teeth have appeared, they should be cleansed with a very soft brush, using either one of the preceding solutions, or a suitable dentifrice. The following, recommended by Zsigmondy, gives excellent results:

R. Magnesii carb

Pulv. saponis......................aa drachm iiss.

Pulv. ossis sepiæ........ ............drachm iiss.

Essentiæ menthæ......................gtt. iv.—M.

In very young children the following may be better:

R. Magnesii carb......................aa gr. lxxv.

Cretæ præparat.,

Sodii salicylatis.......................drachm iij gr. xiv.

Essentiæ menthæ.....................gtt. iv.—M.

When the milk teeth commence to decay they should be filled at once, that they may be preserved as long as possible.—*Dental Record.*

Dr. Chas. T. Parks of Chicago, says, all anæsthetics are dangerous, and all of them occasionally cause death, and the most astounding part about it to outside individuals is, that doctors can not tell why the patients die under these circumstances. These cases of death under anæsthetics are always unexpected, and in the vast majority of cases unavoidable.

